# Next-Generation Sequencing Combined with Specific PCR Assays To Determine the Bacterial 16S rRNA Gene Profiles of Middle Ear Fluid Collected from Children with Acute Otitis Media

**DOI:** 10.1128/mSphere.00006-17

**Published:** 2017-03-22

**Authors:** Saara Sillanpää, Lenka Kramna, Sami Oikarinen, Markku Sipilä, Markus Rautiainen, Janne Aittoniemi, Jussi Laranne, Heikki Hyöty, Ondrej Cinek

**Affiliations:** a Department of Otorhinolaryngology and Head and Neck Surgery, Tampere University Hospital and School of Medicine, University of Tampere, Tampere, Finland; b Department of Pediatrics, 2nd Faculty of Medicine, Charles University in Prague and University Hospital Motol, Prague, Czech Republic; c Department of Virology, School of Medicine, University of Tampere, Tampere, Finland; d Fimlab Laboratories, Tampere, Finland; University of Nebraska Medical Center

**Keywords:** 16S profiling, acute otitis media, bacteriome profiling, mass sequencing, next-generation sequencing

## Abstract

The aim of the study was to analyze the bacteriome of acute otitis media with a novel modification of next-generation sequencing techniques. Outpatient children with acute otitis media were enrolled in the study, and middle ear fluids were collected during 90 episodes from 79 subjects aged 5 to 42 months (median age, 19 months). The bacteriome profiles of middle ear fluid samples were determined by a nested-PCR amplification of the 16S rRNA gene (V4 region), followed by mass sequencing. The profiling results were compared to the results of specific PCR assays targeting selected prevalent pathogens. Bacteriome profiling using nested amplification of low-volume samples was aided by a bioinformatic subtraction of signal contaminants from the recombinant polymerase, achieving a sensitivity slightly lower than that of specific PCR detection. *Streptococcus pneumoniae* was detected in 28 (31%) samples, *Haemophilus influenzae* in 24 (27%), *Moraxella catarrhalis* in 18 (20%), *Staphylococcus* spp. in 21 (23%), *Turicella otitidis* in 5 (5.6%), *Alloiococcus otitidis* in 3 (3.3%), and other bacteria in 14 (16%) using bacteriome profiling. *S. pneumoniae* was the dominant pathogen in 14 (16%) samples, *H. influenzae* in 15 (17%), *M. catarrhalis* in 5 (5.6%), *T. otitidis* in 2, and *Staphylococcus auricularis* in 2. Weaker signals of *Prevotella melaninogenica*, *Veillonella dispar*, and *Veillonella montpellierensis* were noted in several samples. Fourteen samples (16%) were not explainable by bacterial pathogens; novel causative agents were not detected. In conclusion, unbiased bacteriome profiling helped in depicting the true mutual quantitative ratios of ear bacteria, but at present, its complicated protocol impedes its routine clinical use.

**IMPORTANCE** Although *S. pneumoniae*, *H. influenzae*, and *M. catarrhalis* have been long established as the most important pathogens in acute otitis media using culture and specific PCR assays, the knowledge of their mutual quantitative relations and possible roles of other bacteria is incomplete. The advent of unbiased bacteriome 16S rRNA gene profiling has allowed the detection of nearly all bacteria present in the sample, and it helps in depicting their mutual quantitative ratios. Due to the difficulties in performing mass sequencing in low-volume samples, only a few bacteriome-profiling studies of otitis media have been published, all limited to cases of chronic otitis media. Here, we present a study on samples obtained from young children with acute otitis media, successfully using a strategy of nested PCR coupled with mass sequencing, and demonstrate that the method can confer quantitative information hardly obtainable by other methods.

## Abstract (precis)

Although *S. pneumoniae*, *H. influenzae*, and *M. catarrhalis* have been long established as the most important pathogens in acute otitis media using culture and specific PCR assays, the knowledge of their mutual quantitative relations and possible roles of other bacteria is incomplete. The advent of unbiased bacteriome 16S rRNA gene profiling has allowed the detection of nearly all bacteria present in the sample, and it helps in depicting their mutual quantitative ratios. Due to the difficulties in performing mass sequencing in low-volume samples, only a few bacteriome-profiling studies of otitis media have been published, all limited to cases of chronic otitis media. Here, we present a study on samples obtained from young children with acute otitis media, successfully using a strategy of nested PCR coupled with mass sequencing, and demonstrate that the method can confer quantitative information hardly obtainable by other methods.

## INTRODUCTION

According to traditional bacterial culture, *Haemophilus influenzae*, *Streptococcus pneumoniae*, and *Moraxella catarrhalis* have been established as the main causes of acute otitis media (AOM) (1, 2). The detection of bacterial species by culture can, however, be biased by the various growth properties of the agents, and therefore, the popularity of molecular testing is growing: it can provide more exact information about the bacterial etiology of AOM and has also been instrumental in the systematic detection of novel candidate organisms, such as *Alloiococcus otitidis* (3).

Since AOM evolves relatively fast and results from an acute inflammatory process, the presence of any bacterium in high quantity in the middle ear fluid (MEF) is generally accepted as a sign of its causative role in that AOM episode. However, causality is less clear for bacterial species that are found in lower quantities in the MEF. Occasionally, viruses are the causative agents, and the continuous feed of nasopharyngeal flora through the eustachian tube during the inflammatory process may mislead the bacteriological assessment. Furthermore, the external ear canal may contaminate the sample, depending on the way the MEF is collected upon myringotomy. Others (3–11) and we (12) have performed studies with specific PCR detection assays for MEF bacteria in AOM: although the studies differed in the definition of cases, in the spectrum of tested pathogens, and in the PCR primers and protocols used, they mostly agreed on the high frequencies and quantities of *H. influenzae* and *S. pneumoniae*, whereas the frequencies of other bacterial species have varied widely between the studies. Bacteriome-profiling methods were therefore warranted for obtaining an unbiased picture of the bacterial flora and to discover as-yet-unidentified bacteria (7).

Mass sequencing has made it possible to characterize the whole bacteriome by parallel profiling of the 16S rRNA gene in the whole bacterial population. Theoretically, its unbiased character allows the detection of nearly all bacteria present in the sample, their taxonomic evaluation, and mutual relative quantification. This makes it possible to obtain more detailed information on pathogens in AOM and to identify microbes whose etiological role could have remained dubious using traditional microbe-specific methods.

However, the application of mass sequencing in research on otitis media has proven difficult, and only three studies of otitis media based on 16S rRNA gene profiling have been published to date (13–15), all of them on chronic otitis media. The paucity of published studies has clearly demonstrated the technical difficulty in the amplification of low-quantity samples with primers carrying indices and adapters for the mass sequencing.

The goal of the present study was to characterize the bacterial composition of MEF from young Finnish children with AOM and assess the role of pathogens not established in the disease etiology. For this purpose, we adapted a protocol of sensitive nested PCR coupled with mass sequencing, capable of characterizing bacterial 16S rRNA gene profiles in samples with small quantities of bacteria.

## RESULTS

### Samples and their bacterial profiles.

Ninety MEF samples obtained during AOM episodes were collected from 79 children aged 5 to 42 months (median age, 19 months). Eleven children contributed two samples during two separate AOM episodes.

**FIG 1:**
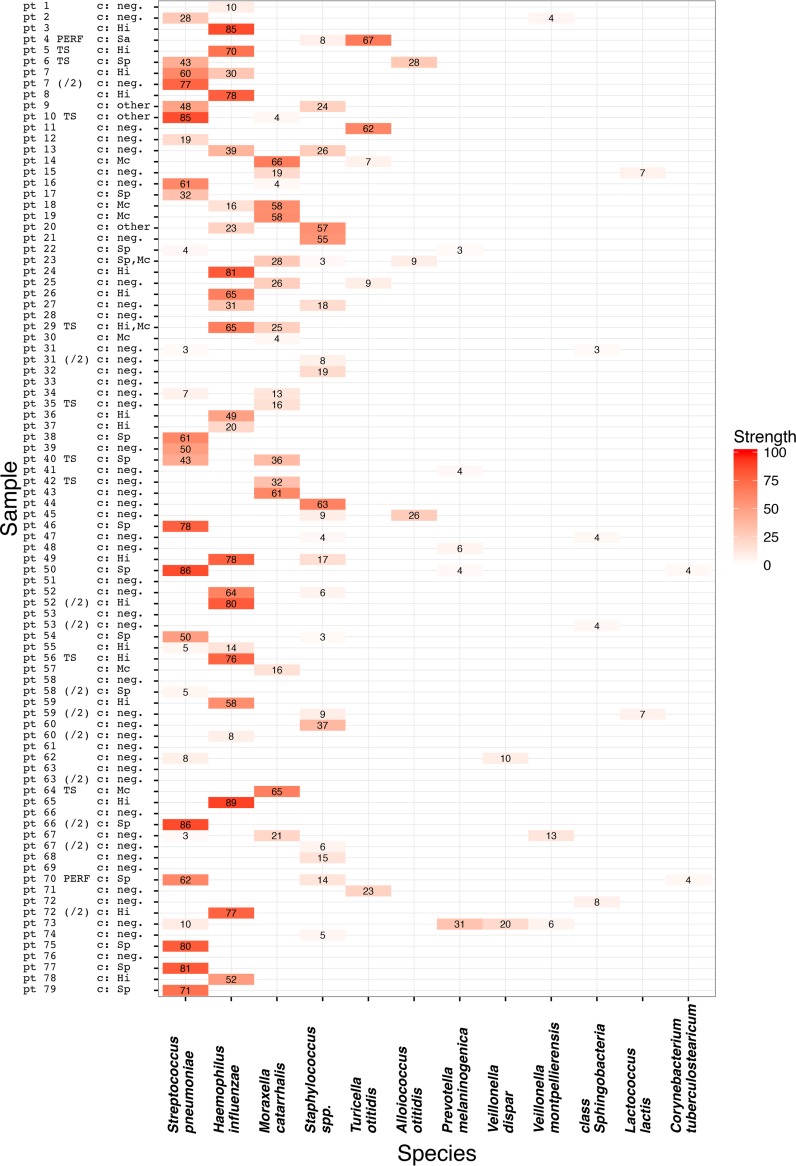
Bacteria found in AOM samples and strengths of their signals. The vertical axis shows individual samples: /2, second sample from the subject; PERF, sample from perforation; TS, sample from tympanostomy tube; other samples are from myringotomy; c, culture; neg., culture negative; Sp, *S. pneumoniae*; Hi, *H. influenzae*; Mc, *M. catarrhalis*; Sa, *Staphylococcus aureus*; other, other bacteria. The horizontal axis shows the nine most abundant species or genera that exceeded 3% of the total sample sequencing signal in at least two AOM samples. The intersections are the relative abundances of the total sequencing signal as percentages (rounded to the nearest integer). Taxa comprising <3% of the total sample sequencing signal were disregarded. The assignation of species by 16S rRNA gene profiling is simplified, since the 16S profiling of the V4 region could not distinguish between closely related species in several instances, as follows. (i) *S. pneumoniae* and the less frequent *S. pseudopneumoniae*; the latter is commonly (mis)identified as *S. pneumoniae* by clinical microbiology laboratories worldwide. The sequence of the profiled V4 region of the 16S rRNA gene is also closely related to those of several other streptococci. (ii) *H. influenzae* and the less frequent *H. haemolyticus*; the latter could be excluded in culture-positive cases by its beta hemolysis. (iii) *M. catarrhalis* and the less frequent *Moraxella nonliquefaciens*; the two could be differentiated only by classic microbiological techniques, including differences in typical antibiograms. Finally, (iv) the 16S profiles in the V4 region are identical in many *Staphylococci*; please see the text for methods that disentangled the signals.

**FIG 2:**
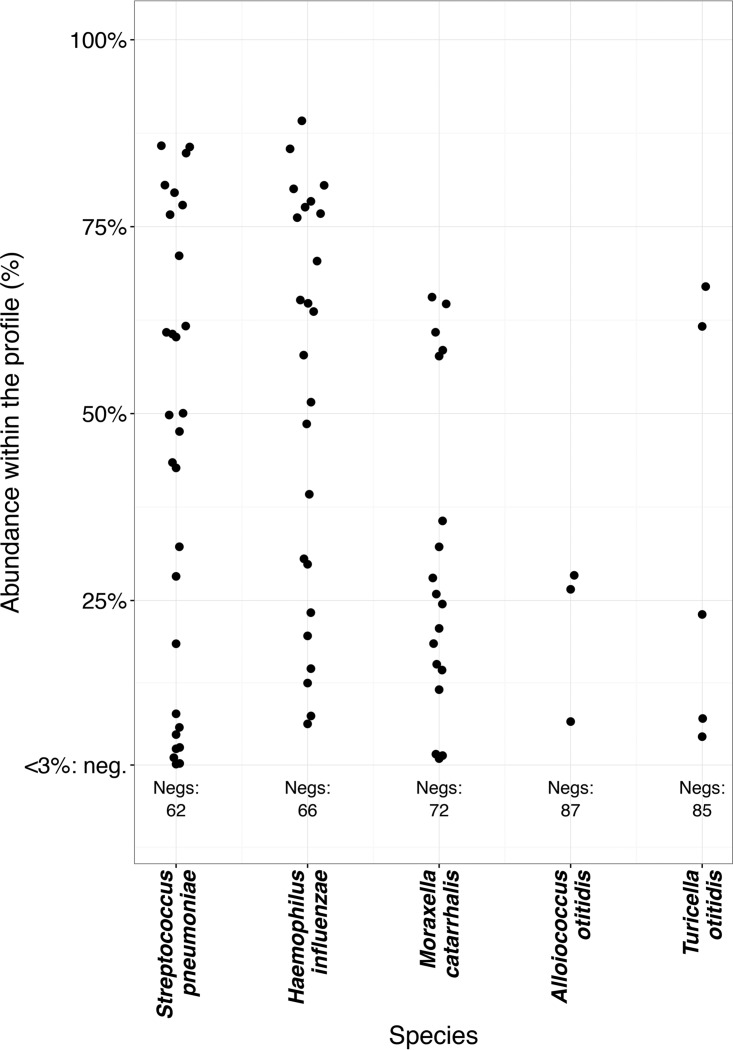
Quantities of the bacteria assessed using the proportions within the individual sample profiles. Negs, count of samples that were negative for the bacterium in the 16S profiling, i.e., had a quantity lower than 3% of the profile signal.

The compositions of the 16S rRNA gene profiles of the MEF samples are shown in Fig. 1, along with the culture results. The relative presence and relative abundance of different bacteria are shown in Table 1 and Fig. 2. The 16S rRNA gene signal of an individual taxon in a sample was expressed as the fraction of the overall signal from that sample. Bacteria in individual samples were quantified into four categories, as follows: (i) the dominant pathogen (half or more of the sequencing signal within a sample), (i) the sole finding, with less than half of the signal (the rest of the signal being contaminant signal from the recombinant polymerase), (iii) a nondominant part of a mixed flora (the bacterium being assigned less than half of reads and present with others in the sample), and (iv) negative (no reads or less than 3% of the signal within the sample).

**TABLE 1:** Bacteria found in the 16S profiles

Finding in the bacterial profile^a^	No. of samples positive for the species (*n* = 90)	
	No. of positive samples	% of all samples
*Streptococcus pneumoniae*	28	31
As a dominant pathogen^b^	14	16
Sole finding but <50% of signal^c^	3	3.3
Nondominant part of mixed flora^d^	11	12

*Haemophilus influenza*	24	27
As a dominant pathogen	15	17
Sole finding but <50% of signal	3	3.3
Nondominant part of mixed flora	6	6.7

*Moraxella catarrhalis*	18	20
As a dominant pathogen	5	5.6
Sole finding but <50% of signal	4	4.4
Nondominant part of mixed flora	9	10

*Staphylococcus* spp.	21	23
As a dominant pathogen	3	3.3
Sole finding but <50% of signal	6	6.7
Nondominant part of mixed flora	12	13

*Turicella otitidis*	5	5.6
As a dominant pathogen	2	2.2
Sole finding but <50% of signal	1	1.1
Nondominant part of mixed flora	2	2.2

*Alloiococcus otitidis*	3	3.3
As a dominant pathogen	0	0
Sole finding but <50% of signal	0	0
Nondominant part of mixed flora	3	3.3

Other bacteria not listed above	14	16
As a dominant pathogen	0	0
Sole finding but <50% of signal	3^e^	3.3
Nondominant part of mixed flora	11^f^	12

No clear bacterial finding	14	16
No bacterium found	11	12
Undetermined species, <5% of signal	3	3.3

a The bacteria originating from the PCR components (*Taq* polymerase) are not shown.

b A dominant pathogen was defined as a bacterium that makes up half or more of the total 16S rRNA gene profile.

c Bacterium occupying 3.0 to 49% of the sequencing signal; no other bacteria were detectable over the threshold 3.0% signal except the contaminant signal from *Taq* polymerase.

d Bacterium occupying 3 to 49% of the sequencing signal; also, other bacteria were present in the profile at >3.0%.

e *Prevotella melaninogenica* (4% in sample from patient 41 and 6% in sample from patient 48) and undetermined *Sphingobacterium* (8% in patient 72). All three samples were taken by myringotomy.

f *Prevotella melaninogenica* (31% of the profile of sample from patient 73, 3% in patient 22, and 4% in patient 50), *Veillonella dispar* (20% in patient 73 and 10% in patient 72), *Veillonella montpellierensis* (13% in patient 67, 6% in patient 73, and 4% in patient 2), *Lactococcus lactis* (7% in patient 15 and 7% in second sample from patient 59), *Corynebacterium tuberculostearicum* (4% from patient 50 and 3% from patient 70, both in samples with dominant *S. pneumoniae*), and undetermined *Sphingobacterium*.

The most frequently observed species was *S. pneumoniae*, being present in 28 (31%) samples, in half of which it showed a strong, dominant signal. The second most frequently observed pathogen was *H. influenzae* (24 samples [27%]), also frequently dominant. Simultaneous strong positivity of these two agents was observed only once in our data set (*S. pneumoniae* and *H. influenzae* in sample 1 of patient 7) (Fig. 1). *M. catarrhalis* was present in 18 (20%) profiles, most often as a part of mixed flora in rather small quantities. However, in five samples (5.6%), it was a clearly dominant pathogen with few or no other bacteria present.

*Staphylococcus* spp. were frequent as a genus (21 samples [23%]) but were seldom the dominant pathogen (3 samples). The variable region 4 (V4) 16S rRNA gene profiles were not instrumental in further taxonomic classification of staphylococci, as the sequences of V4 are identical for numerous *Staphylococcus* species. From the previous testing, we knew that only one of the samples was positive for *S. aureus* among those with *Staphylococcus* as dominant pathogen (patient 44) (Fig. 1). We therefore performed Sanger sequencing of the V3 to V5 regions for the remaining two samples with dominant *Staphylococcus* signals (patients 20 and 21) (Fig. 1) and found strong signals for *Staphylococcus auricularis*; this bacterium was also found in smaller quantities in several other samples by specific PCR.

### Bacteria less often implicated in AOM etiology.

*Turicella otitidis* was observed in five samples (5.6%), of which two occurrences were a strongly positive dominant finding, one was a weaker signal from a sole bacterium present in the sample, and two came from mixed flora. *A. otitidis* was found in three samples (3.3%), always as a component of polymicrobial flora: once with *H. influenzae* (patient 6), once with *M. catarrhalis* and a *Staphylococcus* sp. (patient 23), and once with a *Staphylococcus* sp. (patient 45).

No other bacteria were noted as strong dominant pathogens, but upon inspection of the weaker signals, we found and confirmed *Prevotella melaninogenica*, *Veillonella dispar*, and *Veillonella montpellierensis*, mostly as a component of a multibacterial flora (Fig. 1).

No candidate for a causative agent was found in 14 samples (16%), either because there remained no bacterial signal after subtraction of contaminant signal arising from PCR chemicals (11 instances) or because such a signal was weak and could not be unambiguously taxonomically assigned (3 instances).

### Comparison with specific PCR.

**FIG 3:**
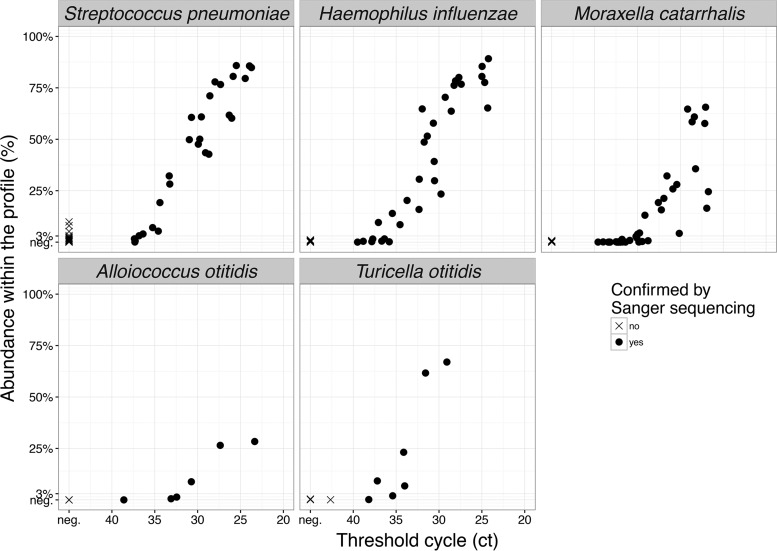
Comparison of detection by specific real-time PCR and by 16S profiling. Horizontal axis, threshold cycle of the respective specific PCR; vertical axis, proportion of the overall profiling signal within the sample. Note that the V4 sequence of *Streptococcus pneumoniae* is nearly identical to those of several further streptococci (e.g., *Streptococcus dentisani*, *Streptococcus tigurinus*, *Streptococcus oralis*, *Streptococcus mitis*, and *Streptococcus infantis*). These are most likely present in several samples, denoted by crosses along the vertical axis of the top left panel: here, the pneumococcus-specific real-time PCR test using the autolysin gene (*lytA*) is negative, but the weak signal in 16S profiling indicates the presence of these streptococci.

The positive results of 16S profiling showed very good agreement with the results of specific PCR tests performed previously for *H. influenzae*, *S. pneumoniae*, *M. catarrhalis*, and *A. otitidis* (12). The PCR testing was done before this mass sequencing was performed and was therefore blinded to the profiling results. The comparison is plotted in Fig. 3. The agreement between the profiling and specific PCR quantification was especially tight when the pathogen was dominant. The negativity of a bacterium in the 16S profile, however, was not an entirely reliable indicator of its true absence: very small quantities of bacteria often remained undetected by 16S profiling, yet they were still positive in specific PCR tests (e.g., *M. catarrhalis* with a PCR signal beyond cycle 35). Furthermore, the use of variable region V4 of the 16S rRNA gene did not allow detailed determination of the species of several genera of important pathogens, so accurate species identification was inferred from specific PCR tests we had performed before (12).

## DISCUSSION

The present work demonstrates that only a limited repertoire of bacteria can be deemed responsible for the majority of pediatric AOM cases. The three main causative bacteria, *S. pneumoniae*, *H. influenzae*, and *M. catarrhalis*, are complemented by the less prevalent *T. otitidis*, *A. otitidis*, and *S. auricularis*. The bacteriome profiling sets the bacteria in a mutual quantitative context and, thus, contrasts species with a likely etiological contribution to other species present in much smaller quantities.

Although we observed no novel dominant causative agents, our results may contribute to the knowledge of species only rarely seen in AOM. First, *T. otitidis* was a clearly dominant pathogen in two samples and the sole abundant bacterium in another sample. So far, the discussion of whether this agent is a colonizer from the outer ear canal or a causative pathogen has not been resolved (16)—our results suggest that in some AOM cases, it could be a true causative agent. Second, another such causative agent may be *S. auricularis*, which was present in high abundance in two samples in children without tympanostomy tubes. The bacterium was first described in 1983 by Kloos and Schleifer (17), who found it to be an important component of the flora of the external auditory canal. Despite several anecdotal reports on severe infections with this bacterium (e.g., see references 18 and 19), it has been mostly regarded only as a cause of otitis externa (20). Although we cannot exclude that both of our findings of *S. auricularis* represent massive inadvertent contaminations from the external auditory canal, the large quantity in the MEF may rather point toward genuine replication in the middle ear.

The proportion of *A. otitidis* in our set of AOM samples was lower than in some other earlier studies, and this organism’s signal was seen exclusively as part of polymicrobial infections. The bacterium was the most prevalent one in the 16S rRNA gene profile in one sample only, but even then, two other organisms were clearly present. Although *A. otitidis* has attracted considerable attention in the literature since it was first reported in otitis media (21), we are afraid that some of the later reports may have overestimated its frequency and abundance, possibly due to technical limitations of the PCR assays used therein. While earlier works correctly utilized molecular assays that confirmed the correctness of the product by accurately determining its length on polyacrylamide gels or verified its character by using melting analysis, sequencing, or ligation reaction (5, 22, 23), some of the later studies relied solely only on agarose gel electrophoresis without any verification of the inner sequence of the product (3, 4, 24–27); some of these detection results might be incorrect. Under less stringent conditions, the primers (22) tend toward cross-reaction with human DNA, yielding a fragment of 238 bases (chr2:233,742,816–233,743,053 in the GRCh38 assembly), whose size is usually indistinguishable from the amplicon size of *A. otitidis* (261 bases) in agarose gel electrophoresis.

A previous study by Smith-Vaughan et al. has suggested that the summed abundance of three major pathogens detected by specific PCRs in AOM is much lower than the total bacterial load estimated by another assay (7). The authors called for the use of bacteriome-profiling methods as a possible solution to the discrepancy, because they assumed that a large proportion of the total bacterial load might be comprised of as-yet-unidentified bacteria. Most likely, this is not the case. First, as the authors stated, instead of using MEF samples collected under sterile conditions, they had to resort to testing ear discharge samples, which may have been strongly contaminated with ear canal flora. Second, the difference may be an artifact caused by, e.g., the mutual difference in efficacies of amplification of the four primer pairs used for specific PCR detection. Finally, the signal from the recombinant polymerase might inflate the total bacterial load in low-abundance samples. In contrast, our approach offers quite accurate characterization of the 16S profiles, because the set of contaminant species that originated from *Taq* polymerase has been characterized and subtracted, incidentally providing a kind of internal quantitative standard. Thus, the present study was able to characterize the whole spectrum of bacteria, and still, the previously described pathogens constituted the majority of the profiles without any support for the existence of a major unknown bacterial causative agent.

The sensitivity of 16S rRNA gene profiling stands between the low sensitivity of culture and the high sensitivity of specific PCR assays targeted to individual organisms. This can be seen from the results of the present study and is especially pronounced for *M. catarrhalis*. Here, the sensitivity of specific PCR clearly superseded that of 16S profiling. Regarding theoretical considerations of the potential of 16S profiling as a diagnostic tool, although it is rather sensitive in the present modification, it could not be relied upon as a primary diagnostic tool in a situation where the disease is mostly caused by a limited set of several well-known agents with available specific PCR tests. We also observed many small-quantity findings in the 16S profiles where it has not been clarified whether they reflect true biological significance of the agent: indeed, the DNA profiling may detect dead bacteria, although studies in chinchillas have shown that bacterial DNA in MEF disappears within 3 days of bacterial cell death (28). Moreover, innocuous bacteria may passively enter the middle ear during viral infection and can be detected in small quantities during AOM caused by viral pathogens. In addition, some of the bacteria detected with sequencing might reflect the normal flora of the middle ear (15). In instances with positive culture, the identified organisms were detected by 16S rRNA gene profiling as well, with the single exception of a sample that was positive for *S. pneumoniae* by culture and specific PCR. That sample was negative for *S. pneumoniae* in the 16S profile, while three other organisms were detected.

The literature on 16S profiling in otitis media is scanty and limited to chronic otitis media only: Liu et al. analyzed one patient with chronic otitis media (13), and Jervis-Bardy et al. analyzed 11 children with otitis media with effusion using one round of PCR and failed to obtain sufficient signals in half of the samples (14). Recently, Neeff et al. utilized nested PCR in the characterization of bacteriome profiles in 24 predominantly adult patients with chronic suppurative otitis media (71% with cholesteatoma) and 22 healthy control ears (15). Thus, the present study is a significant contribution to the field, being the largest 16S profiling study of otitis media and the only one of AOM so far. We obtained a clear 16S signal from the majority of MEF samples and were able to confirm most of our findings by specific PCR. Together with the work by Neeff et al. (15), we demonstrated that nested PCR is necessary for 16S amplification of MEF. While Neeff et al. terminated the nested amplification before a false signal from the *Taq* polymerase could emerge, we used much longer amplification with fully developed false signal in negative controls, which was then subtracted from the signals for real samples. This new modification solved the well-known problem of samples with low bacterial content—the ribosomal nucleic acid coming from the *Taq* polymerase in the PCRs competes with the true signal coming from the sample (29, 30). We optimized this method to achieve the lowest possible level of contaminating background signal using a strategy inspired by Spangler et al. (31), and similar to their results, the optimal chemistry found was HotStar polymerase (Qiagen). An additional advantage of computational subtraction of the polymerase-derived signal is that a stable low level of contamination serves as an excellent internal control in 16S profiling. The relative quantity of any ear bacterium may then serve as a guide to indicate the relevance of a pathogen.

The middle ear is sterile according to specific PCR tests and bacterial cultures (32). However, Neeff et al. detected bacteria with mass sequencing of samples from healthy adults’ middle ears in up to 43% of cases (15). They showed that bacterial loads in the healthy middle ear and mastoid cavity are low. They detected small amounts of species from genera like *Novosphingobium*, *Staphylococcus*, *Streptococcus*, *Escherichia-Shigella*, and *Burkholderia*. Of those bacteria, only *Staphylococcus* species were detected in our study, and in 20% of the samples, it was seen in low abundance, which might reflect its role as part of the normal microbiota of the middle ear or as a contaminant from the outer ear canal. These studies, however, cannot answer questions about normal pediatric middle ear flora.

This study has several important technological strengths. First, our protocol was able to provide reliable signals from the majority of the 90 samples, including the low-abundance samples. The use of triplicate reactions helped substantially to ensure a homogenous signal, which could then be used for profiling. Because of the relative quantification that is a characteristic of the 16S profiling, we were able to distinguish dominant pathogens present in large quantities from the signals of bacteria present in small quantities whose pathogenic participation might be unlikely. The existence of a stable background arising from the rRNA gene contaminating the recombinant polymerase served as an exogenous internal control of amplification and a competitive PCR target. We verified and confirmed all prominent signals from the 16S profiling by means of specific real-time PCR from the original samples. In most cases, we used yet another level of verification by performing Sanger sequencing of the ensuing PCR products. This degree of certainty is, to our knowledge, rather exceptional among studies of bacteria in AOM.

One limitation of the described method is its complicated protocol: for clinical use, a considerable daily count of samples would be needed to make the 16S sequencing cost effective. This generally renders the method unsuitable for routine clinical practice, where early antibiotic response is desirable. In addition, the V4 region of the 16S rRNA gene cannot discriminate exact species of several clinically relevant otopathogens. Namely, *Haemophilus influenzae* has a V4 sequence identical to that of *H. haemolyticus*, which is nonencapsulated (i.e., nontypeable) and generally considered nonpathogenic. Furthermore, *Streptococcus pneumoniae* has a V4 sequence identical to that of *Streptococcus pseudopneumoniae*, and even more importantly, nearly identical to those of several other streptococci, and therefore, the pneumococcus-specific PCR tests, including ours, have long used the autolysin gene (*lytA*) rather than the poorly informative 16S rRNA gene. Also, numerous species of the genus *Staphylococcus* are indiscriminate in the V4 region. Specific PCR assays are thus needed for such agents, but for surprisingly many organisms, including those of potential clinical relevance, no published primer sequences are known.

In conclusion, our work has shown the composition of microbial middle ear flora in AOM in children, excluded the possibility of a large gap between the known agents and the total bacterial load, and demonstrated that 16S profiling by mass sequencing can confer information hardly obtainable by other methods.

## MATERIALS AND METHODS

### Patients and their MEF samples.

Children were enrolled at the Department of Otorhinolaryngology, Tampere University Hospital, Tampere, Finland, between September 2010 and December 2011. The diagnosis of AOM was based on the presence of MEF with signs of inflammation of the tympanic membrane, or alternatively, otorrhea through a tympanostomy tube or a spontaneous perforation of the tympanic membrane and symptoms of acute respiratory infection.

The MEF specimens were collected after myringotomy with a sterile suction tip. In children with tympanostomy tubes or spontaneous perforations of the eardrum, MEF specimens were obtained from the middle ear by suction. The sample set was identical to what had been described in our previous study (12). The study protocol was approved by the Ethical Committee of the Tampere University Hospital (reference number R10026), and written informed consent was obtained from all participating families.

### Processing of the samples, culture, and pathogen-specific PCR.

**FIG 4:**
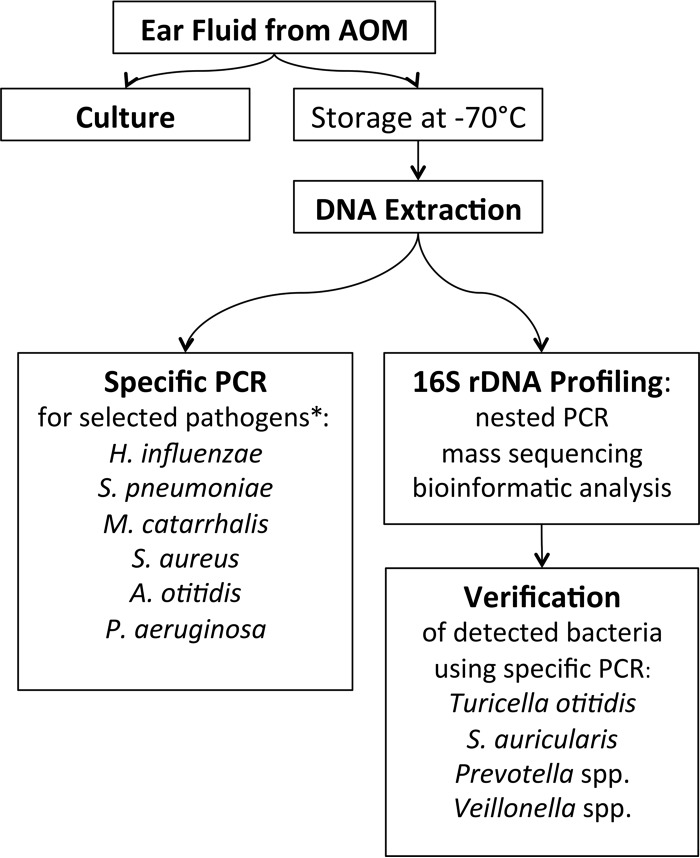
Workflow of the study. *, data from specific PCR tests of these pathogens come from our previous study (12).

The workflow diagram of sample processing is shown in Fig. 4. One aliquot of each MEF sample was obtained for bacterial culture, and another aliquot was immediately frozen and stored at −70°C until DNA extraction, specific PCR tests, and nested 16S rRNA gene mass-sequencing profiling were performed. Bacterial culturing, the extraction of nucleic acids, and pathogen-specific PCR for six candidate pathogens (*H. influenzae*, *A. otitidis*, *M. catarrhalis*, *S. pneumoniae*, *Pseudomonas aeruginosa*, and *Staphylococcus aureus*) have been described previously (12). Bacterial cultures were performed aerobically. The result was listed as negative if no bacterial growth was seen. Other flora consisted of atypical bacteria found in minute quantities. In our previous study (12), we tested the present sample set using semiquantitative real-time PCR assays for *H. influenzae*, *A. otitidis*, *M. catarrhalis* (the three assays were targeted to the 16S rRNA gene with primers by Holder et al. [24] and a probe previously used by Nadkarni et al. [33]), *S. pneumoniae* (34), *Pseudomonas aeruginosa* (35), and *Staphylococcus aureus* (36).

### Profiling of the bacterial populations using next-generation sequencing of the 16S rRNA gene.

Due to the character of MEF from acute otitis media in children, the total bacterial load in most samples was low, and reliable microbiome profiling was not achievable by only a single round of PCR (data not shown). We therefore designed a nested PCR amplification protocol for the V4 region of the 16S rRNA gene, followed by next-generation sequencing of the second-round products.

We first preamplified the region of interest using primers flanking the 16S rRNA gene variable regions V3 and V5: 2 μl of DNA was added to a 15-µl reaction mixture containing 1× HotStar *Taq* buffer, a total concentration of 3.0 mM MgCl_2_, 0.1 mM each deoxynucleoside triphosphate (dNTP), 670 nM primers 16S_V3F (CCTACGGGAGGCAGCAG [37]) and 16S_V5R (CCCGTCAATTCMTTTRAGT [38]), 200 nM fluorescent TaqMan probe “16S_probe” (CGTATTACCGCGGCTGCTGGCAC [33]), and 0.4 unit Qiagen HotStar *Taq* polymerase (buffer, magnesium, and polymerase; Qiagen, Hilden, Germany). All chemicals and consumables whose character allowed it (water, buffers, and magnesium solution) were first irradiated in a UV DNA cross-linker using an energy of 5 J; this step should fragment or eliminate possible contaminating DNA.

The first round of amplification was performed with 15 min of initial denaturation and polymerase unblocking at 95°C and a given number of cycles consisting of 15 s of denaturation at 94°C, 1 min of annealing with data collection at 60°C, and 1 min of synthesis at 72°C. Every sample and control was run in three replicates that differed by the length of amplification in this first round of PCR: 25, 30, and 35 cycles. The positive controls were five serial dilutions of the mock community (BEI Resources), and the negative controls were positions with extracts from sterile water instead of MEF; the controls were amplified by these three programs, being irregularly interspersed among the clinical samples.

The PCR products of the first round were then diluted 1:9 with UV-irradiated PCR water and subjected to the second round of PCR. Here, we used indexed primers flanking variable region V4 of the 16S rRNA gene according to the method of Kozich et al. (39). The primers carried adapters enabling next-generation sequencing and indices that distinguished the samples from each other. The reaction mixture contained 1× HotStar *Taq* buffer, a total concentration of 3.0 mM MgCl_2_, 0.1 mM each dNTP, 500 nM indexed forward and reverse primer, probe “16S_V4_IN_PROBE” to assess the amplification (6-carboxyfluorescein [FAM]-CATTYCACCGCTACAC-dark quencher, minor groove binder; own design), 0.5 U HotStar *Taq* polymerase (Qiagen, Hilden, Germany), and 2 µl of the diluted product of the first round (thus, every sample was run in three replicates differing by the number of first-round cycles). The program consisted of 15 min at 95°C, 30 cycles of 15 s of denaturation at 94°C, 1 min of annealing and data collection at 60°C, and 30 s of synthesis at 72°C. Several randomly chosen positions were checked on an agarose gel.

The set of reactions included negative controls of the whole process (18 positions with PCR water added either into the DNA extraction or at various levels of the downstream process), as well as positive controls of the PCR. We utilized 330 of the total of 384 possible index combinations, irregularly positioning the replicates of individual samples into the primer index grid. The remaining positions were left empty, but data were collected in order to assess false signals.

The products of the second PCR round, i.e., indexed libraries, were first purified by using AMPure magnetic beads (Beckman Coulter, Inc., Fullerton, CA) added to the product in an 8:10 ratio. Next, we diluted the purified libraries with water at a 1:10,000 ratio and quantified them by using the KAPA library quantification kit (Kapa Biosystems, Wilmington, MA) that targets the sequencing adapters and, thus, quantifies only fragments amenable to next-generation sequencing. Using these quantitation data, the libraries were equalized to a single concentration of 2 nM and pooled. The pool was mixed with 25% PhiX DNA (Illumina), an addition necessary for the MiSeq sequencing instrument (Illumina) in order to compensate for an unwanted homogeneity of fluorescence signal generated by conserved regions of the PCR products. After denaturation, the pool was sequenced on an Illumina MiSeq instrument using a 2 × 250 base-sequencing kit (Illumina).

### Bioinformatic processing of the mass sequencing data and elimination of signal arising from the recombinant polymerase.

After demultiplexing the reads, the *mothur* suite version 1.25.0 (40) was used to merge, trim, and filter the reads. The sequences were then transferred with a custom Python script into the *Qiime* suite, version 1.9.1 (41), where we removed chimeras by using *usearch* version 5.2.236 (42), merged replicates of the same sample, and clustered the sequences into operational taxonomic units (OTUs) based on a similarity of 97% or more. For each OTU, the most abundant sequence was selected as a representative of the respective OTU. Sequences were identified and taxonomically classified using the Silva database version 108 (43), and the assignment of the 150 most frequent ones was also manually curated using the National Center for Biotechnology Information (NCBI) 16S database.

The signals observed in the profiling consisted of bacteria present in the ear fluid and of the artifacts arising most likely from the residual DNA in the recombinant *Taq* polymerase. To eliminate the artifacts, we utilized the profiles obtained from negative controls processed in the same sequencing run: such signals helped to identify which bacterial species were contaminants from the polymerase and to subtract their OTUs from the analysis of the ear fluid samples. There were several distinct signals present in the negative-control profiles in approximately constant mutual ratios, all originating from bacteria predominantly occurring in the environment and the prevalent ones never before implicated in human disease (see Fig. S1 in the supplemental material).

10.1128/mSphere.00006-17.1 FIG S1  Bacterial DNA found in negative controls. The overall number of 6 no-template controls (water instead of MEF subjected to extraction and all subsequent analytic steps) was expanded into 17 control positions in the second round of amplification. Data presented here are collapsed back into the six original samples. Download FIG S1, PDF file, 0.1 MB. Copyright © 2017 Sillanpää et al. 2017 Sillanpää et al. This content is distributed under the terms of the Creative Commons Attribution 4.0 International license.

The efficacy of the 16S rRNA gene profiling was assessed using the standard mock community, whose analysis was run in 18 replicates also differing by the number of PCR cycles in the first PCR round. The instances of these reactions were randomly scattered across the sample index map.

After subtraction of the contaminant signal arising from reaction mixture components, we filtered the ensuing OTUs for minimum read count (>20) and minimum occurrence (>3 samples). Then, the reads were normalized to 10,000 per sample and OTUs ordered by the total count of normalized reads; taxa were retained if they represented over 3% of the signal in at least two samples and otherwise were removed. Finally, taxa were tabulated at the levels of genera and species against the sample identifiers. The table was manually inspected, and relevant species verified or further taxonomically sorted using specific PCR and Sanger sequencing.

### Performance measures of 16S mass sequencing.

The sequencing yielded 8 million reads, of which 59% were aligned to the *PhiX* phage: the addition of this phage DNA is necessary for technical reasons inherent to the MiSeq sequencer. Among individual index combinations (replicates), the count of 16S rRNA gene reads ranged from 593 to 18,000 (median 3,460; interquartile range [IQR], 2,272 to 4,675), with the exception of six instances where amplification failed. Consequently, six samples were analyzed in duplicates, whereas the rest were analyzed in triplicates.

Nineteen of 21 species in the control mock community were correctly identified, with the exceptions of *Staphylococcus epidermidis*, which could be classified only to the level of genus by using the V4 region (a known problem with the resolution ability of the V4 region), and of* Propionibacterium acnes*, which was not detected, most likely due to terminal mismatches of both primers to its sequence.

Eighteen no-template control positions with amplified water were randomly scattered throughout the map of index combinations. Because we let the signal originating from the residual DNA in the recombinant polymerase develop fully in these negative controls, we consistently observed a profile consisting of strong signals from *Acidovorax* and related genera and weaker signals from *Pelomonas*, *Ralstonia*, *Undibacterium*, *Sphingobium*, *Sphingomonas*, and several other bacterial genera (see Fig. S1 in the supplemental material). These taxa (OTUs) originating from the *Taq* polymerase were subtracted and disregarded in the downstream analysis of ear fluid samples.

### Verification of selected signals from 16S profiling.

In addition to the real-time quantification data obtained in the previous study (*S. pneumoniae*, *H. influenzae*, *M. catarrhalis*, *Alloiococcus otitidis*, *Staphylococcus aureus*, and *Pseudomonas aeruginosa* [12]), the results of the 16S profiling stimulated the need for specific confirmatory PCR tests for verification of further OTUs. Specifically, these were OTUs containing *T. otitidis*, *Veillonella* spp., *Prevotella* spp., and *Staphylococcus auricularis*. The tests were real-time PCRs with a hydrolysis TaqMan probe annealing within the V4 region, whose primers were designed from an alignment of all observed OTUs from our 16S profiling (see Table S1 in the supplemental material). The reactions were specific within the limited repertoire observed in the present study plus the strains of the control mock community, but it should be noted that these reactions mostly lack absolute specificity within the whole kingdom of *Bacteria* (Silva Test Prime webpage [44]). The PCR products of positive samples were confirmed by bidirectional Sanger sequencing.

10.1128/mSphere.00006-17.2 TABLE S1  Primers used for verification of selected OTUs found in 16S profiles from the present study. Download TABLE S1, PDF file, 0.05 MB. Copyright © 2017 Sillanpää et al. 2017 Sillanpää et al. This content is distributed under the terms of the Creative Commons Attribution 4.0 International license.

### Accession number(s).

The sequencing data, along with clinical and laboratory metadata, have been deposited in the Sequence Read Archive (NCBI) under GenBank accession number SRP079688 (BioProject no. PRJNA327935).

## References

[1] Ruohola A, Meurman O, Nikkari S, Skottman T, Salmi A, Waris M, Österback R, Eerola E, Allander T, Niesters H, Heikkinen T, Ruuskanen O 2006 Microbiology of acute otitis media in children with tympanostomy tubes: prevalences of bacteria and viruses. Clin Infect Dis 43:1417–1422. doi:10.1086/509332. 17083014 PMC7107988

[2] Casselbrant ML, Mandel EM, Kurs-Lasky M, Rockette HE, Bluestone CD 1995 Otitis media in a population of black American and white American infants, 0-2 years of age. Int J Pediatr Otorhinolaryngol 33:1–16. doi:10.1016/0165-5876(95)01184-D. 7558637

[3] Harimaya A, Takada R, Hendolin PH, Fujii N, Ylikoski J, Himi T 2006 High incidence of Alloiococcus otitidis in children with otitis media, despite treatment with antibiotics. J Clin Microbiol 44:946–949. doi:10.1128/JCM.44.3.946-949.2006. 16517881 PMC1393137

[4] Kaur R, Adlowitz DG, Casey JR, Zeng M, Pichichero ME 2010 Simultaneous assay for four bacterial species including Alloiococcus otitidis using multiplex-PCR in children with culture negative acute otitis media. Pediatr Infect Dis J 29:741–745. doi:10.1097/INF.0b013e3181d9e639. 20335823 PMC3581301

[5] Marsh RL, Binks MJ, Beissbarth J, Christensen P, Morris PS, Leach AJ, Smith-Vaughan HC 2012 Quantitative PCR of ear discharge from Indigenous Australian children with acute otitis media with perforation supports a role for Alloiococcus otitidis as a secondary pathogen. BMC Ear Nose Throat Disord 12:11. doi:10.1186/1472-6815-12-11. 23033913 PMC3546424

[6] Palmu AAI, Saukkoriipi PA, Lahdenkari MI, Kuisma LK, Makela PH, Kilpi TM, Leinonen M 2004 Does the presence of pneumococcal DNA in middle-ear fluid indicate pneumococcal etiology in acute otitis media? J Infect Dis 189:775–784. doi:10.1086/381765. 14976593

[7] Smith-Vaughan HC, Binks MJ, Marsh RL, Kaestli M, Ward L, Hare KM, Pizzutto SJ, Thornton RB, Morris PS, Leach AJ 2013 Dominance of Haemophilus influenzae in ear discharge from Indigenous Australian children with acute otitis media with tympanic membrane perforation. BMC Ear Nose Throat Disord 13:12. doi:10.1186/1472-6815-13-12. 24099576 PMC3852835

[8] Stol K, Verhaegh SJC, Graamans K, Engel JAM, Sturm PDJ, Melchers WJG, Meis JF, Warris A, Hays JP, Hermans PWM 2013 Microbial profiling does not differentiate between childhood recurrent acute otitis media and chronic otitis media with effusion. Int J Pediatr Otorhinolaryngol 77:488–493. doi:10.1016/j.ijporl.2012.12.016. 23369612 PMC7132406

[9] Virolainen A, Salo P, Jero J, Karma P, Eskola J, Leinonen M 1994 Comparison of PCR assay with bacterial culture for detecting Streptococcus pneumoniae in middle ear fluid of children with acute otitis media. J Clin Microbiol 32:2667–2670. 7852553 10.1128/jcm.32.11.2667-2670.1994 PMC264139

[10] Xu Q, Kaur R, Casey JR, Adlowitz DG, Pichichero ME, Zeng M 2011 Identification of Streptococcus pneumoniae and Haemophilus influenzae in culture-negative middle ear fluids from children with acute otitis media by combination of multiplex PCR and multi-locus sequencing typing. Int J Pediatr Otorhinolaryngol 75:239–244. doi:10.1016/j.ijporl.2010.11.008. 21126776 PMC3563323

[11] Yatsyshina S, Mayanskiy N, Shipulina O, Kulichenko T, Alyabieva N, Katosova L, Lazareva A, Skachkova T, Elkina M, Matosova S, Shipulin G 2016 Detection of respiratory pathogens in pediatric acute otitis media by PCR and comparison of findings in the middle ear and nasopharynx. Diagn Microbiol Infect Dis 85:125–130. doi:10.1016/j.diagmicrobio.2016.02.010. 26971180 PMC7126741

[12] Sillanpää S, Oikarinen S, Sipilä M, Kramna L, Rautiainen M, Huhtala H, Aittoniemi J, Laranne J, Hyöty H, Cinek O 2016 Moraxella catarrhalis might be more common than expected in acute otitis media in young Finnish children. J Clin Microbiol 54:2373–2379. doi:10.1128/JCM.01146-16. 27413187 PMC5005485

[13] Liu CM, Cosetti MK, Aziz M, Buchhagen JL, ContenteCuomo TL, Price LB, Keim PS, Lalwani AK 2011 The otologic microbiome: a study of the bacterial microbiota in a pediatric patient with chronic serous otitis media using 16SrRNA gene-based pyrosequencing. Arch Otolaryngol Head Neck Surg 137:664–668. doi:10.1001/archoto.2011.116. 21768410

[14] Jervis-Bardy J, Rogers GB, Morris PS, Smith-Vaughan HC, Nosworthy E, Leong LEX, Smith RJ, Weyrich LS, De Haan J, Carney AS, Leach AJ, O’Leary S, Marsh RL 2015 The microbiome of otitis media with effusion in Indigenous Australian children. Int J Pediatr Otorhinolaryngol 79:1548–1555. doi:10.1016/j.ijporl.2015.07.013. 26228497

[15] Neeff M, Biswas K, Hoggard M, Taylor MW, Douglas R 2016 Molecular microbiological profile of chronic suppurative otitis media. J Clin Microbiol 54:2538–2546. doi:10.1128/JCM.01068-16. 27487953 PMC5035421

[16] von Graevenitz A, Funke G 2014 Turicella otitidis and Corynebacterium auris: 20 years on. Infection 42:1–4. doi:10.1007/s15010-013-0488-x. 23775360

[17] Kloos WE, Schleifer KH 1983 Staphylococcus auricularis sp. nov.: an inhabitant of the human external ear. Int J Syst Bacteriol 33:9–14. doi:10.1099/00207713-33-1-9.

[18] Lew SQ, Saez J, Whyte R, Stephenson Y 2004 Peritoneal dialysis-associated peritonitis caused by Staphylococcus auricularis. Perit Dial Int 24:195–196. 15119644

[19] Hoffman DJ, Brown GD, Lombardo FA 2007 Early-onset sepsis with Staphylococcus auricularis in an extremely low-birth weight infant—an uncommon pathogen. J Perinatol 27:519–520. doi:10.1038/sj.jp.7211773. 17653219

[20] Roland PS, Stroman DW 2002 Microbiology of acute otitis externa. Laryngoscope 112:1166–1177. doi:10.1097/00005537-200207000-00005. 12169893

[21] Faden H, Dryja D 1989 Recovery of a unique bacterial organism in human middle ear fluid and its possible role in chronic otitis media. J Clin Microbiol 27:2488–2491. 2808673 10.1128/jcm.27.11.2488-2491.1989 PMC267063

[22] Hendolin PH, Markkanen A, Ylikoski J, Wahlfors JJ 1997 Use of multiplex PCR for simultaneous detection of four bacterial species in middle ear effusions. J Clin Microbiol 35:2854–2858. 9350746 10.1128/jcm.35.11.2854-2858.1997 PMC230074

[23] Hendolin PH, Paulin L, Ylikoski J 2000 Clinically applicable multiplex PCR for four middle ear pathogens. J Clin Microbiol 38:125–132. 10618075 10.1128/jcm.38.1.125-132.2000 PMC86036

[24] Holder RC, Kirse DJ, Evans AK, Peters TR, Poehling KA, Swords WE, Reid SD 2012 One third of middle ear effusions from children undergoing tympanostomy tube placement had multiple bacterial pathogens. BMC Pediatr 12:87. doi:10.1186/1471-2431-12-87. 22741759 PMC3475091

[25] Aydın E, Taştan E, Yücel M, Aydoǧan F, Karakoç E, Arslan N, Kantekin Y, Demirci M 2012 Concurrent assay for four bacterial species including Alloiococcus otitidis in middle ear, nasopharynx and tonsils of children with otitis media with effusion: a preliminary report. Clin Exp Otorhinolaryngol 5:81–85. doi:10.3342/ceo.2012.5.2.81. 22737288 PMC3380117

[26] Harimaya A, Takada R, Somekawa Y, Fujii N, Himi T 2006 High frequency of Alloiococcus otitidis in the nasopharynx and in the middle ear cavity of otitis-prone children. Int J Pediatr Otorhinolaryngol 70:1009–1014. doi:10.1016/j.ijporl.2005.10.012. 16310863

[27] Holder RC, Kirse DJ, Evans AK, Whigham AS, Peters TR, Poehling KA, Swords WE, Reid SD 2015 Otopathogens detected in middle ear fluid obtained during tympanostomy tube insertion: contrasting purulent and non-purulent effusions. PLoS One 10:e0128606. doi:10.1371/journal.pone.0128606. 26039250 PMC4454603

[28] Post JC, Aul JJ, White GJ, Wadowsky RM, Zavoral T, Tabari R, Kerber B, Doyle WJ, Ehrlich GD 1996 PCR-based detection of bacterial DNA after antimicrobial treatment is indicative of persistent, viable bacteria in the chinchilla model of otitis media. Am J Otolaryngol 17:106–111. 8820185 10.1016/s0196-0709(96)90005-8

[29] Lupan I, Ianc MB, Ochis C, Popescu O 2013 The evidence of contaminant bacterial DNA in several commercial Taq polymerases. Rom Biotechnol Lett 18:8007–8012.

[30] Niimi H, Mori M, Tabata H, Minami H, Ueno T, Hayashi S, Kitajima I 2011 A novel eukaryote-made thermostable DNA polymerase which is free from bacterial DNA contamination. J Clin Microbiol 49:3316–3320. doi:10.1128/JCM.00584-11. 21775543 PMC3165589

[31] Spangler R, Goddard NL, Thaler DS 2009 Optimizing Taq polymerase concentration for improved signal-to-noise in the broad range detection of low abundance bacteria. PLoS One 4:e7010. doi:10.1371/journal.pone.0007010. 19753123 PMC2737620

[32] Westerberg BD, Kozak FK, Thomas EE, Blondel-Hill E, Brunstein JD, Patrick DM 2009 Is the healthy middle ear a normally sterile site? Otol Neurotol 30:174–177. doi:10.1097/MAO.0b013e31819225a0. 19060773

[33] Nadkarni MA, Martin FE, Jacques NA, Hunter N 2002 Determination of bacterial load by real-time PCR using a broad-range (universal) probe and primers set. Microbiology 148:257–266. doi:10.1099/00221287-148-1-257. 11782518

[34] Carvalho Mda G, Tondella ML, McCaustland K, Weidlich L, McGee L, Mayer LW, Steigerwalt A, Whaley M, Facklam RR, Fields B, Carlone G, Ades EW, Dagan R, Sampson JS 2007 Evaluation and improvement of real-time PCR assays targeting lytA, ply, and psaA genes for detection of pneumococcal DNA. J Clin Microbiol 45:2460–2466. doi:10.1128/JCM.02498-06. 17537936 PMC1951257

[35] De Vos D, Lim AJ, Pirnay JP, Struelens M, Vandenvelde C, Duinslaeger L, Vanderkelen A, Cornelis P 1997 Direct detection and identification of Pseudomonas aeruginosa in clinical samples such as skin biopsy specimens and expectorations by multiplex PCR based on two outer membrane lipoprotein genes, oprI and oprL. J Clin Microbiol 35:1295–1299. 9163432 10.1128/jcm.35.6.1295-1299.1997 PMC229737

[36] Pichon B, Hill R, Laurent F, Larsen AR, Skov RL, Holmes M, Edwards GF, Teale C, Kearns AM 2012 Development of a real-time quadruplex PCR assay for simultaneous detection of nuc, Panton-Valentine leucocidin (PVL), mecA and homologue mecALGA251. J Antimicrob Chemother 67:2338–2341. doi:10.1093/jac/dks221. 22687894

[37] Muyzer G, de Waal EC, Uitterlinden AG 1993 Profiling of complex microbial populations by denaturing gradient gel electrophoresis analysis of polymerase chain reaction-amplified genes coding for 16S rRNA. Appl Environ Microbiol 59:695–700. 7683183 10.1128/aem.59.3.695-700.1993 PMC202176

[38] Jaric M, Segal J, Silva-Herzog E, Schneper L, Mathee K, Narasimhan G 2013 Better primer design for metagenomics applications by increasing taxonomic distinguishability. BMC Proc 7(Suppl 7):S4. doi:10.1186/1753-6561-7-S7-S4. PMC4044206 24564926

[39] Kozich JJ, Westcott SL, Baxter NT, Highlander SK, Schloss PD 2013 Development of a dual-index sequencing strategy and curation pipeline for analyzing amplicon sequence data on the MiSeq Illumina sequencing platform. Appl Environ Microbiol 79:5112–5120. doi:10.1128/AEM.01043-13. 23793624 PMC3753973

[40] Schloss PD, Westcott SL, Ryabin T, Hall JR, Hartmann M, Hollister EB, Lesniewski RA, Oakley BB, Parks DH, Robinson CJ, Sahl JW, Stres B, Thallinger GG, Van Horn DJ, Weber CF 2009 Introducing mothur: open-source, platform-independent, community-supported software for describing and comparing microbial communities. Appl Environ Microbiol 75:7537–7541. doi:10.1128/AEM.01541-09. 19801464 PMC2786419

[41] Caporaso JG, Kuczynski J, Stombaugh J, Bittinger K, Bushman FD, Costello EK, Fierer N, Peña AG, Goodrich JK, Gordon JI, Huttley GA, Kelley ST, Knights D, Koenig JE, Ley RE, Lozupone CA, McDonald D, Muegge BD, Pirrung M, Reeder J, Sevinsky JR, Turnbaugh PJ, Walters WA, Widmann J, Yatsunenko T, Zaneveld J, Knight R 2010 QIIME allows analysis of high-throughput community sequencing data. Nat Methods 7:335–336. doi:10.1038/nmeth.f.303. 20383131 PMC3156573

[42] Edgar RC 2010 Search and clustering orders of magnitude faster than BLAST. Bioinformatics 26:2460–2461. doi:10.1093/bioinformatics/btq461. 20709691

[43] Quast C, Pruesse E, Yilmaz P, Gerken J, Schweer T, Yarza P, Peplies J, Glöckner FO 2013 The SILVA ribosomal RNA gene database project: improved data processing and web-based tools. Nucleic Acids Res 41:D590–D596. doi:10.1093/nar/gks1219. 23193283 PMC3531112

[44] Klindworth A, Pruesse E, Schweer T, Peplies J, Quast C, Horn M, Glöckner FO 2013 Evaluation of general 16S ribosomal RNA gene PCR primers for classical and next-generation sequencing-based diversity studies. Nucleic Acids Res 41:e1. doi:10.1093/nar/gks808. 22933715 PMC3592464

