# Supplementary material for: Next-Generation Sequencing Combined with Specific PCR Assays To Determine the Bacterial 16S rRNA Gene Profiles of Middle Ear Fluid Collected from Children with Acute Otitis Media
Source: mSphere. 2017 Mar 22;2(2):e00006-17. doi: 10.1128/mSphere.00006-17 (PMC5362748; doi:10.1128/mSphere.00006-17)

Supplemental Figure 1. Bacterial DNA found in negative controls

The overall number of 6 no-template controls (water instead of MEF subjected to extraction and all subsequent analytic steps) were expanded into 17 controls positions in the second round of amplification. Data presented here are collapsed back into the six original samples.

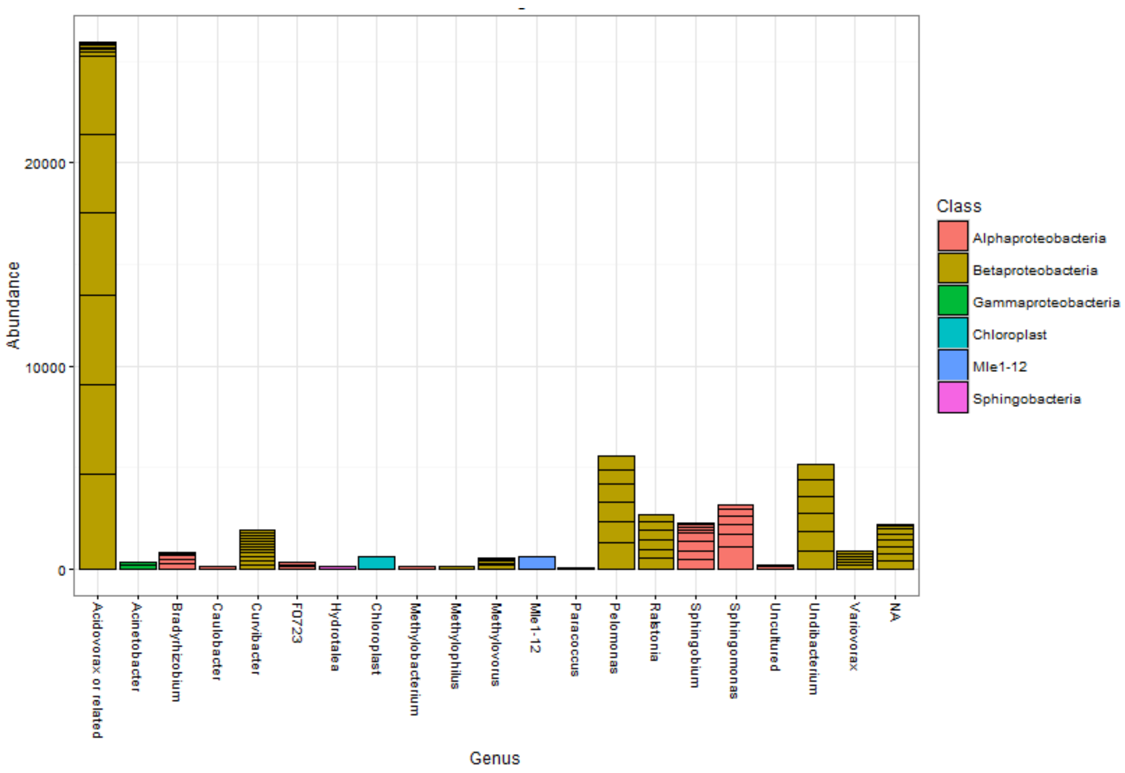

Supplement: FIG S1 [file sph002172256sf2.pdf]
