# Supplementary material for: Next-Generation Sequencing Combined with Specific PCR Assays To Determine the Bacterial 16S rRNA Gene Profiles of Middle Ear Fluid Collected from Children with Acute Otitis Media
Source: mSphere. 2017 Mar 22;2(2):e00006-17. doi: 10.1128/mSphere.00006-17 (PMC5362748; doi:10.1128/mSphere.00006-17)
Supplement: TABLE S1 [file sph002172256st1.pdf]

SUPPLEMENTARY MATERIALS

Supplemental Table 1. Primers used for verification of selected OTUs found in 16S profiles from the present study.

**Verified organism or OUT**	**Forward primer, reverse primer**
Turicella otitidis[(a)]	GCGTCGTCTGTGAAAGTCTG<br>ATCGGTGTTCCTCCTGATATCTG
Prevotella, OTU 11[(a)]	GGCGTTATCCGGATTTATTGGGTT<br>CGTCAGTAACGTTACGGTAAGCT
Prevotella, OTU 14[(a)]	GGCGTTATCCGGATTTATTGGGTT<br>TCAGTTGCGCTCCCGTCAGCT
Prevotella, OTU 98[(a)]	GGCGTTATCCGGATTTATTGGGTT<br>GTCAGTTGCAGCCCGGACAC
Prevotella, OTU 134[(a)]	GGCGTTATCCGGATTTATTGGGTT<br>GCGTCAGTTGTGCTCCCGTAA
Veillonella, all its OTUS[(a)]	AACCCCGTGATGGGATGGAA<br>GCGTCAGTTTTCGTCCAGAAAG
Veillonella dispar[(a)]	GCGCGCGCAGGCGGATTGGTC<br>GCGTCAGTTTTCGTCCAGAAAG
Veillonella other species[(a)]	GCGCGCGCAGGCGGCCTATCC<br>GCGTCAGTTTTCGTCCAGAAAG
Staphylococcus auricularis[(b)]	GTTCTACAGTGAAAGGCGGCT<br>GAAGACTCTATCTCTAGAGCG

Own design of primers. These primers were intended for verification and quantification of OTUs of interest within the repertoire of bacteria in the present study, plus the control Mock Community. The primers therefore lack absolute specificity within the kingdom of *Bacteria* and their use for general detection of the organisms is very strongly discouraged.

(a) These reactions use a degenerate MGB probe that provides an approximation of the bacterial quantity of the sample. (16S_V4_IN_PROBE, FAM - CATTYCACCGCTACAC - dark quencher, MGB).

(b) The primers are theoretically specific for *S. auricularis* but the specificity is maintained mainly by the forward primer. As only several mismatches prevent cross-reactivity with *S. warneri, pasteuri* or *pulmonis*, it may be expected at less stringent conditions, or high concentration of the agens.
